# The social return on investment of physical activity and nutrition interventions—a scoping review

**DOI:** 10.3389/fspor.2023.1296407

**Published:** 2024-01-08

**Authors:** Anna Stielke, Kathryn Ashton, Andrew Cotter-Roberts, Mariana Dyakova

**Affiliations:** WHO Collaborating Centre on Investment for Health and Well-Being, Public Health Wales NHS Trust, Cardiff, United Kingdom

**Keywords:** social value, social return on investment, public health, physical activity, nutrition, review

## Abstract

**Introduction:**

Scarcity of resources and mounting pressures on health systems make it critical to evaluate new and existing public health interventions related to physical activity and nutrition. The Social Return on Investment (SROI) framework has gained prominence for capturing traditional variables such as economic costs and returns, as well as wider beneficial social and environmental outcomes. A scoping review was conducted to present the existing evidence on the SROI of physical activity and nutrition interventions, demonstrating the wider benefits of these interventions.

**Methodology:**

Existing peer-reviewed evidence and grey literature was collated to identify physical activity and nutrition interventions that were evaluated using the SROI framework between January 1996 up until February 2022. Only literature published in the English language, interventions that were conducted in high-income countries were considered for inclusion. Study information and economic data was entered into a pre-prepared data extraction sheet and eligible studies were quality assessed using a 12-point quality assessment framework for SROI studies.

**Results:**

This review identified a total of 21 SROI studies, with only four deriving from peer-reviewed literature sources. In total, 18 studies evaluated physical activity interventions, one study was purely focused on nutrition, whereas the two remaining studies presented a mix of physical activity and nutrition. The majority of studies derived from the United Kingdom (*n* = 16) with very few of the studies published prior to 2010 (*n* = 1). In total, four studies were classified as low quality based on the 12-point quality assessment framework used for this review. Outcomes of the relevant studies show that the benefits of these interventions have added value to families, communities and the wider environments of the target groups.

**Conclusion:**

This scoping review is adding to research conducted to understand the wider value of public health interventions such as physical activity and nutrition interventions using the SROI framework. This is important so that the development and implementation of public health interventions have the greatest value to people and society, which also benefits decision-makers to effectively and sustainably allocate scarce resources.

## Background

In past decades, there has been a major epidemiological shift from communicable to non-communicable diseases (NCDs) in societies around the world (1). Currently, NCDs are one of the leading causes of morbidity and mortality globally (2), with mortality estimated to account for 74% of all deaths annually (3). NCDs are associated with numerous negative health outcomes, including an elevation of cardiovascular diseases, type 2 diabetes, mental illness and certain types of cancers (4). Major contributing factors for the increase in NCDs are unhealthy dietary pattern and low physical activity levels created by obesogenic environments, especially at workplaces where sedentary work practices have become the norm (5, 6). In 2022, the World Health Organization (WHO) estimated that 1 billion people globally were obese, 39 million of these were children alone with projections indicating an increase in these numbers in the next decade (7). Negative health outcomes of NCDs come with significant direct and indirect economic impacts especially for health systems. The economic burden on countries is substantial with data from 51 countries indicating that around 26% of total health spending is attributable to NCDs (8). For example, it is projected that direct and indirect costs related to obesity in the population will rise as a percentage of Gross Domestic Product (GDP) in the next decades across countries, with for example 4.88% of the GDP associated costs in Thailand in 2060 (9). This increase is estimated to reach a similar scale in low-, middle- and high-income countries. Global prevalence standardised by age of insufficient physical activity was 27.5% in 2016 (10). Countries in the Organisation for Economic Co-operation and Development (OECD) are spending 8.4% of their total health budget on the treatment of obesity-related diseases such as type 2 diabetes (11). For instance, by 2050 the United Kingdom-wide (UK) National Health Service (NHS) costs attributable to overweight and obesity in the population is estimated to be around £9.7 billion (12). Examples of direct costs include treatment services, while indirect costs include productivity loss and absenteeism (13, 14). However, there are also multiple negative implications for the wider system of the individual experiencing excess body weight, for example on relationship building with family members and maintaining social networks, as well as on education and employability (15). Multiple studies also show that obesity is a risk factor for short- and long-term sickness absence rates (14, 16).

Scarcity of resources and mounting pressures on health systems due to challenges such as COVID-19, make it critical to evaluate physical activity and nutrition interventions to understand their wider value. There has also been a noticeable shift of policies towards emphasising the wider impacts of public health interventions. The 2030 Agenda to drive sustainable development on a global level recognises the link between “sustainable development and other relevant ongoing processes in the economic, social and environmental fields” (17). The Tallin Charter from 2008 states that “health systems should recognise investment in health is investment in human development, social well-being and health” (18).

More recently, a move from traditional evaluation methods to understand not just the financial value of such interventions, but also the wider social, environmental and economic value has been observed (19). Traditionally, economic methods such as cost-effectiveness have been used to evaluate public health interventions to determine financial inputs and outputs but do not capture wider social and environmental value (20). However, evidence suggests that public health interventions such as physical activity and nutrition interventions generate broader indirect benefits (21). For example, the World Health Organization's *Global Action Plan on physical activity* (22) states that “in addition to the multiple health benefits of physical activity, societies that are more active can generate additional return on investment including a reduced use of fossil fuels, cleaner air and less congested, safer roads”. This is also evident within a previous review which advocates for and makes the “financial and social” case for investing in sports and recreation services, as they benefit not just overall health, but can have wider implications for example, education and youth crime reduction (23).

The economic evaluation framework of Social Return on Investment (SROI) has gained prominence in the past decade for capturing economic costs and returns, as well as wider social and environmental outcomes (often referred to as “soft” outcomes, outcomes that do not have a market value such as life satisfaction). The framework enables the monetarisation of these outcomes into a singular monetary figure to present a holistic value of interventions which is not captured as part of more traditional economic evaluation methods (24, 25). It helps to try and equate the value that people place on certain benefits, as well as dis-benefits caused by the intervention to other things that they attach importance to in their lives. This indicates that the SROI framework has the ability to measure the broader socio-economic outcomes, analysing views of multiple stakeholders. Presenting a more holistic picture of interventions through the SROI framework has led to a much broader concept of value as the framework allows to capture the wider impact of interventions; a concept often referred to as the “triple bottom line” (26, 27).

A recent systematic review provides an overview of the application of SROI in public health, concluding that the framework is a relevant tool to systematically account for outcomes of an intervention that would have been missed in traditional value for money evaluations (28). This makes the framework, and principles within it, suitable for measuring the value of physical activity and nutrition interventions. However, little published evidence exists which specifically demonstrates the wider value and outcomes of physical activity and nutrition interventions. Building on previously published systematic scoping reviews (29, 30), this unique scoping review aims to explore the SROI evidence base of physical activity and nutrition interventions by outlining information such as distribution across the types of interventions, outcomes as well as SROI ratios amongst others. This will to help make the case for investment in this area of public health.

The findings of this review, in addition to traditional health economic methods, can be used to inform policy-makers, funding agencies and budget holders about the wider value of investing in physical activity and nutrition interventions.

## Methodology

A scoping review was conducted to explore the available evidence base on the use of the SROI framework for physical activity and nutrition interventions. This type of evidence review was chosen as it helps to systematically assess the potential size and scope of the available evidence on a specific thematic area (31).

Peer-reviewed evidence was retrieved from PubMed (32) (which retrieves evidence from other databases such as Medline) a well as Google Scholar, whereas non-peer-reviewed evidence (grey literature) was identified Google Scholar and the following organisational websites: Social Value UK, New Economics Foundation and the World Health Organization. Grey literature was collated using a combination of the following search terms: “overweight/obesity” OR “excess weight” “weight management” and “intervention” OR “program” OR “service” and “social value” OR “social return on investment” with peer-reviewed evidence retrieved using the search string as outlined in the Supplementary Table S1. Grey literature was identified from

Evidence identified in the peer-reviewed and grey literature was used to apply the snowballing principle to detect relevant additional literature for inclusion. The search was conducted by one evidence reviewer; however, an additional reviewer also assessed the identified evidence for inclusion and conflicting opinions were discussed to reduce the risk of bias (33).

All relevant evidence identified from the searches were screened for inclusion, firstly by title and abstract and then by full-text based on the criteria. At the initial search stage, evidence was only considered if it was published in the English language after January 1996 when the SROI framework was developed (34), up until February 2022. At the screening stage, publications were included that were conducted in a high-income country (according to the World Bank classification) (35), provided the SROI of interventions focussed on physical activity and/or nutrition and were available in full excluding for example protocols. At the eligibility stage, evidence was excluded that did not interpret the SROI ratio, meaning data was missing or a description of the economic, social or environmental returns of the identified intervention and the intervention was not relevant to public health (for detailed exclusion criteria see Supplementary Table S2 and please find the complementary inclusion criteria in Table 1).

**Table 1:** Inclusion criteria.

Element	Key concept
Population	Target group focussed on children, adolescence, or adulthood only
Intervention	Public health intervention that specifically focuses on one of the following key themes: weight management, overweight and/or obesity, sedentary behaviour, lifestyle, sports/physical activity, nutrition/diet and were evaluated using the Social Return on Investment (SROI) as framework
Outcome	SROI evaluation showing the social (environmental or economic) return/value created by the intervention identified
Comparison	Not applicable

A pre-developed template was utilised to firstly extract relevant study level information such as description and aim of the intervention, study population and study design. Secondly, economic data was extracted such as outcomes and measurements, SROI results as well as associated measurements for instance attribution, deadweight and drop-off. This review used a 12-point quality assessment framework to determine the quality of included studies, based on the following dimensions, namely: transparency about why the SROI methodology was chosen, documentation of the SROI analysis, study design, precision of the analysis and reflection of the results (Supplementary Table S3). As this review replicates the methodology used for previously published reviews (29) and aims to builds on them, the 12-point quality assessment framework was used rather than other available quality assessment frameworks (36).

## Results

**Figure 1:**
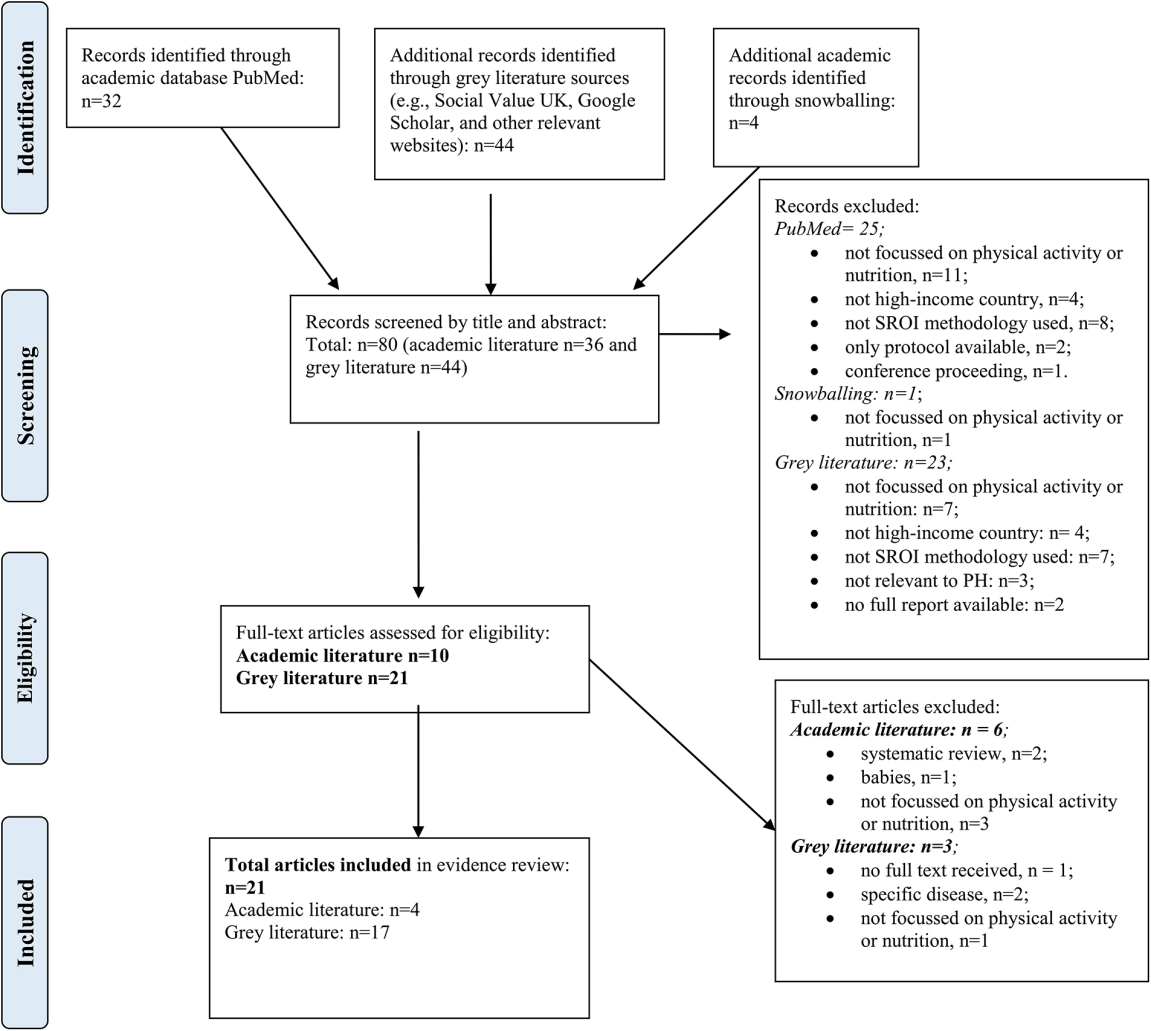
PRISMA flow diagram.

The Preferred Reporting Items for Systematic Review and Meta-Analyses (PRISMA) (37) was used to report findings and identified a total of 21 eligible SROI studies to be included in the final review (Figure 1).

### Summary of study characteristics

Overall, of the 21 studies included in this scoping review, four were retrieved from the academic literature (38–41) and 17 from grey literature sources (42–58). The majority of included studies were published in the UK (*n* = 16, 76.2%), with the remaining studies originating from the Netherlands (*n* = 1; 4.6%), Japan (*n* = 1; 4.6%) and Taiwan (Republic of China; *n* = 3; 14.3%). Only one study which was identified for inclusion was a prospective SROI evaluation which measured the forecasted impact of the specific activity or project at hand (43) with the remainder (*n* = 20) being retrospective SROI evaluations measuring the change that has occurred from the intervention. Very few of the eligible studies were published prior to 2010 (*n* = 1) (53) with the majority of studies published in 2020 (*n* = 5; 23.8%) and 2013 (*n* = 5; 23.8%). In addition, 18 studies fitted into the physical activity category, one study was purely focused on nutrition, whereas the two remaining studies presented a mix of physical activity and nutrition components (Table 1). For the purpose of this scoping review, the target populations addressed in the different studies was split into three age groups, namely childhood and adolescence, adulthood and cross cutting (targeting all age groups). Interestingly, the majority of studies were targeted towards all age groups (*n* = 12; 57.1%), with 23.9% (*n* = 5) of studies targeted towards childhood and adolescence while only 19.0% (*n* = 4) were targeted mainly towards adults (Table 2). In total, four studies scored a low-quality assessment score based on the 12-point quality assessment framework used for this review (59).

**Table 2:** Number of SROI papers per study characteristic and type of intervention.

Study characteristics	Type of intervention			Total
	Physical activity	Nutrition	Cross cutting	
Type of intervention	18	1	2	21
Source of publication				
Academic	3		2	5
Grey	15	1		16
Country				
United Kingdom	15		1	16
The Netherlands			1	1
Taiwan (Republic of China)	2	1		3
Japan	1			1
Main target population				
Childhood/adolescence	3	1	1	5
Adulthood	4			4
Cross cutting	12			12
Year published				
2022	1			1
2021	1			1
2020	3	1	1	5
2019	3			3
2018	1			1
2017	1			1
2014	2			2
2013	5			5
2012	1			1
2009	1			1
Type of SROI				
Forecast/prospective	1			1
Evaluative/retrospective	18	1	1	20
Quality scores				
High (higher or equal 8)	14	1	2	17
Low (lower than 8)	4			4

### Physical activity interventions

In total, 18 SROI studies were identified that focused on physical activity (Table 3), presented through a wide range of different sports. All of the interventions targeted group sport activities, rather than individual sports. Interestingly, four of these interventions had an environmental component to encourage participants to use the natural environment to undertake physical activity (50, 51, 53, 54). SROI ratios for this category of interventions were all positive and ranged from 22.37 to 1.91 per 1 unit invested.

**Table 3:** Social return on investment (SROI) of physical activity interventions.

Reference (ID)	Source of publication	Country	Population	Aim of intervention	Crude SROI	Quality score
Oshimi et al. (38)	Academic	Japan	Childhood/adolescence	Coaches deliver free 30-minute soccer or physical activity lessons to local children in their school.	USD 5.3:1 invested	10
Davies et al. (39)	Academic	United Kingdom	Cross cutting	Provide sports and physical activity across 12 community sport and leisure facilities.	£ Part 1: 1.2:1 invested Part 2: 3.42:1 invested	10
Davies et al. (40)	Academic	United Kingdom	Cross cutting	All physical activities undertaken of all individuals in the sports sector at national level.	£ 1.91:1 invested	10
KPMG Sustainability Consulting Co. (45)	Grey	Taiwan (Republic of China)	Childhood/adolescence	Deliver baseball training programs and activities before and after the baseball competitions.	NT$ 12.49:1 Invested	9
Whitebarn Consulting (42)	Grey	United Kingdom	Cross cutting	Promotion of Gaelic sports games to cater for sporting and social needs in the community.	€ 15.3:1 invested	9
Baker et al. (43)	Grey	United Kingdom	Cross cutting	Encourage a range of community groups, from sports clubs to scout groups and parish and town councils, and schools to in participation in sport and physical activity.	€ 7.25:1 invested	11
Charlton (44)	Grey	United Kingdom	Cross cutting	Help drive improved access to, and an increased participation in, sport and physical activity by giving young people access to free or subsidised coaching in a range of sports	€ 2.85:1 invested	7
Davies et al. (46)	Grey	United Kingdom	Cross cutting	Participating and volunteering in sport and physical activity at national level.	£ 3.28:1 invested	8
Butler and Leatham (47)	Grey	United Kingdom	Children/adolescence	Delivery of physical activity services to young people in challenging areas.	£ 4.21:1 invested	10
ICF GHK Consulting (48)	Grey	United Kingdom	Cross cutting	Providing a wide range of activities on offer in the programme, including judo, golf, tennis, wakeboarding, athletics and free running.	For every £1 invested, the estimates SROI is as follows: 5.50 over 1 year; 7.00 over 3 years; and, 7.50 over 5 years	11
Tilly (49)	Grey	United Kingdom	Cross cutting	Providing a range of physical activities in the sport and Leisure Trust	£ The total of £37.1 million per annum of economic and social gain for MSLT is set against around £5.5million of funding from Manchester Council.^a^	8
New Economics Foundations (50)	Grey	United Kingdom	Cross cutting	Scheme that combines physical activity with local environmental project.	£ 4.02:1 invested	7
Ireland (51)	Grey	United Kingdom	Adulthood	Project that promotes “green” exercise with an awareness raising drive to improve knowledge of the links between nutrition, physical exercise and mental health and wellbeing.	£ 2.02:1 invested	8
Cathay Life Insurance (52)	Grey	Taiwan (Republic of China)	Adulthood	Project inviting adult citizens to walk a set mount of steps each day through incentives.	NT$ 6.30:1 invested	9
O’Neill (53)	Grey	United Kingdom	Cross cutting	Bike path created as part of urban greenspace development to but is being used but a wide range of activities taking place including conservation activities, health walks, mountain bike club, scramble bike up lift and amnesty, community physical activity events etc.	£ 7.63:1 invested	9
Carrick (54)	Grey	United Kingdom	Cross cutting	Led health walks to promote walking opportunities in the area (which include workplace, school and active travelling walks).	£ 8.00:1 invested	9
Davies (55)	Grey	United Kingdom	Cross cutting	Understanding the participation and volunteering of a nation in sport.	£ 2.88:1 invested	7
Parker (56)	Grey	United Kingdom	Cross cutting	Provision of services in a leisure trust including gyms and swimming pools.	£ 22.37:1 invested	4

^a^ SROI is typically presented as a ratio of the value of the benefits achieved per pound spent to achieve those benefits. This may be useful internally to each organisation as a measure of performance relative to prior periods. However, the use of this ratio to compare organisations is inherently flawed due to sector and organisation-specific factors that reduce the level of comparability between organisations. Hence, the results of this report are not presented in the form of a ratio

### Nutrition intervention

The only study identified in this category (57) was targeted at students to support them with financial means to encourage healthy nutrition to eating a regular breakfast (Table 4). This SROI study generated a positive ratio of NT$ 2.20:1 invested.

**Table 4:** Social return on investment (SROI) of nutrition interventions.

Reference (ID)	Source of publication	Country	Population	Aim of intervention	Crude SROI	Quality score
EY Taiwan's Climate Change and Sustainability Services (CCaSS) (57)	Grey	Taiwan (Republic of China)	Childhood/adolescence	Love Breakfast money to serve as students’ breakfast funds during the semester, in hope of enhancing the students’ nutrition, spirits and academic performance.	NT$ 2.20:1 invested	10

### Interventions combining physical activity and nutrition components

Two studies combined physical activity and nutrition components, both of which had a positive SROI ratio. One study focused on children and adolescents exclusively (41) while the other took a more holistic approach targeting families (58) (Table 5).

**Table 5:** Social return on investment (SROI) of cross-cutting interventions.

Reference (ID)	Source of publication	Country	Population	Aim of intervention	Crude SROI	Quality score
Oosterhoff et al. (41)	Grey	The Netherlands	Childhood/adolescence	Providing healthy lunch and daily structured physical activity sessions.	€ 0.01:1 invested The incremental net benefit of HPSF was estimated ate−851/child/2 years (SROI ratio of 0.01)	11
Jones (58)	Grey	United Kingdom	Cross cutting	Providing lifestyle mentoring service (becoming more active, eating healthier) and family weight management service	£ 5.42:1 invested	10

### Outcomes

The eligible studies presented a variety of outcomes beyond just their primary aim of improving physical activity levels and/or dietary behaviour. Studies recorded outcomes for their respective primary target group but also other stakeholders experienced changes due to the respective intervention. Outcomes were diverse in nature and span from hard objective outcomes such as reduction in weight and uptake in exercise to soft outcomes such as social cohesion and happiness. (A full list of all recorded outcomes of all studies is presented in the Supplementary Table S4).

Concerning the physical activity studies (38–40, 43–56) in particular, re-occurring outcomes in all studies were around improvements to mental and physical health. Some of the hard outcomes included improved educational performance, reduction in crime rates, cost savings for the NHS due to improved health and fitness as well as others. All physical activity studies also recorded a variety of soft outcomes such as confidence, with similar outcomes including: sense of identification and belonging as well as self-esteem, an increased sense of satisfaction and achievement, improved discipline and competence as well as improved relations with family members.

With regards to the single nutrition intervention (57), notable outcomes for the primary target group were around schoolchildren's maintenance of health, enhancement of learning, self-exploration and development and improvement of life skills. For other stakeholders some of the recorded outcomes were as follows: parental stress relief and reduction in stress for teachers as well as increased income of community economics and increased connection of community resources for schools and local communities.

The two interventions that combined physical activity and nutrition elements also recorded a variety of outcomes (41, 58). Hard outcomes included: an increase in families health quality of life (HRQOL), levels of absenteeism from school as well as reduced GP and primary care nurse consultations. Some of the soft outcomes included: opportunities for parents to engage in work and other activities increased feeling of happiness and increase in confidence levels.

## Discussion

There is increasing evidence on the use of the SROI framework to evaluate public health interventions (28, 60). In past decades the SROI methodology has received more attention within the literature. The framework has the potential to demonstrate the holistic value of interventions and can capture the many indirect impacts interventions can have on the target group but also other beneficiaries (24, 28).

Other more traditional economic evaluation methods do not capture and quantify soft and hard outcomes alike. Therefore, this scoping review particularly focused on interventions using the SROI framework to understand the wider value provided by nutrition and physical activity interventions. Conducting a scoping review allowed the exploration of the potential size and scope of the available evidence of studies using the SROI framework for physical activity and nutrition related interventions. Based on the outcomes identified in this review, there appears to be a wider value of physical activity and nutrition interventions, this complements and updates particularly the findings of a previously conducted review (30) which focused on identifying physical activity and sport interventions only using the SROI framework. It is also adding to an earlier published review that mapped evidence that used the SROI framework for public health interventions across stages the life course (29).

The majority of physical activity or nutrition intervention studies which were identified through this scoping review derived from grey literature sources, with only four out of 21 of the studies being from peer-reviewed journals. This aligns with a previously conducted literature search (29, 61) on health interventions using the SROI framework which identified a total of 434 with only 107 out of these being academically published (25). This suggests that the SROI framework has primarily been used by third sector organisations too, for example, advocate for continued or new funding (62) compared to academic institutions. There has also been critique especially from the academic environment regarding the accuracy and robustness of the SROI methodology, indicating the low number of studies identified in academic journals. A common discussion point has been around how the proxy measures have been chosen to calculate the ratio and how the ratio has been understood and used; this is a general observation for the SROI framework not specifically to physical activity and nutrition interventions (25, 63, 64). Another noteworthy observation is around the fact that 16 out of the 21 identified studies were conducted in the United Kingdom, this can be due to the fact that this review was only searching for studies published in the English language but also due to the introduction of the Public Services (Social Value) Act 2012 (65) which now requires certain public bodies to consider their impact on the economic, social and environmental well-being of their procurement decisions. Similar to this review, previously published research indicated that most health-related SROI studies derive from Englisch speaking contexts such as the UK and Ireland (61). In addition, it is interesting to note that very little evidence was found regarding the use of SROI as a framework to evaluate specifically nutrition interventions. This might be related to the fact that physical activity and nutrition interventions as well as other public health interventions might still be evaluated using more traditional health economics methods such as cost-benefit analysis opposed to frameworks such as SROI (20).

The majority of the identified studies was evaluative in nature, with only one study being prospective. One reason for this may be that the SROI framework is often used to apply for continued funding and make the case to capture wider value of a certain interventions to inform decision-making processes (24). The majority of interventions are still evaluated using more traditional economic methods such as cost-benefit analysis.

This review applied a 12-point quality assessment framework (59) also used by previously published reviews of SROI evidence (29, 66). However, it must be acknowledged that another, academically published, SROI quality assessment framework is available (36). Looking into the quality scores for the identified studies, one of the noteworthy observations is that the majority of the studies scored high with only a total of four studies scoring low, indicating that most studies are of an acceptable standard to potentially guiding policy and decision makers. However, this does not mean that the studies scoring lower do not convey relevant information. Consistent with the findings of the meta-analysis establishing the 12-point quality assessment framework, studies identified in this review scored particularly low on criteria such as control groups and ex-ante—ex-post observation which may be due to limited resources (59).

## Limitations

While the methodology used for this review (31) is suitable for its aim and followed previously published reviews as part of a series (29, 66), some limitations can be noted. Although this study is not a systematic literature review, the methodology followed was able to give an idea of the scope of the available evidence and a snapshot of eligible studies. Some of the potentially eligible evidence might have been missed and not captured by the search terminology used for this review if evidence was published under a title that was not associated with the search terms used. In addition, only a few databases were used to search for eligible literature, relevant evidence might have been missed. Most SROI studies were derived from grey literature sources, however, there is no single grey literature database for SROI studies specifically focussed on public health interventions such as physical activity and nutrition and some of the relevant studies therefore might have been missed as part of the review. Another important factor to note is that there are studies that use SROI as a framework that have multiple components as part of their intervention which is often referred to as lifestyle interventions (67). These have physical activity and nutrition components and might have been relevant too, but the focus of this review was purely on physical activity and/or nutrition as the main intervention component. Additionally, this review did not aim to assess or compare interventions or for that matter SROI ratios.

Results from this scoping review can be used to understand the value of physical activity and nutrition interventions better and potentially inform its development as well as investment decisions towards these types of interventions. Consequently, this review has the potential to encourage researchers, practitioners and policy- and decision-makers to use SROI as a framework to understand the holistic value of physical activity and nutrition interventions they develop, implement and fund.

## Further research

Results from this scoping review can be used as a baseline. However, further research could be conducted to understand whether other methods are appropriate to understand the holistic value of such interventions. It is also important to further understand how the findings could be appropriately used by funders as well as advocates, decision-makers or those working in the physical activity and nutrition sector to help build the case for continued and new investment in those interventions. Due to public health interventions often being multicomponent interventions consisting of elements such as physical activity and nutrition, it could be beneficial to conduct a review and further research focussing on multi-component public health interventions. Due to the sparsity in the academic literature, it is suggested to increase publications in academic journals should be a focus which not just generates and expands evidence to further the framework for public health related intervention but also improve the quality of publication due to peer-review processes. The review concluded that more research was needed to increase the application of the SROI framework to interventions to showcase and promote the value of the methodology for this particular type of interventions.

## Conclusion

This scoping review is adding to the evidence base on the evaluation of physical activity and/or nutrition interventions using more holistically focused methods such as the SROI framework. SROI as an evaluative tool helps demonstrate that these interventions have a wider value usually not measured through traditional health economics methodologies. In total, this review has identified 21 studies that have measured the social value of physical activity and nutrition related interventions by using the SROI framework; with little evidence that derived from the academic literature. The majority of reports included in this review were on physical activity interventions. This scoping review suggests that physical activity and/or nutrition interventions have a positive social value and therefore wider value for direct and indirect beneficiaries. These can range from improved educational performance, reduced social isolation and improved resilience and self-esteem. It becomes increasingly important that the holistic impact of nutrition and physical activity interventions and programmes is understood so that interventions which have the greatest value to people can be developed and implemented. This also benefits to effectively, equitably and sustainably allocate scarce resources.
