# Supplementary material for: The social return on investment of physical activity and nutrition interventions—a scoping review
Source: Front Sports Act Living. 2024 Jan 8;5:1296407. doi: 10.3389/fspor.2023.1296407 (PMC10801155; doi:10.3389/fspor.2023.1296407)
Supplement: Supplementary file 1 [file Datasheet1.pdf]

Supplementary material

Manuscript title: The Social Return on Investment of physical activity and nutrition interventions – a systematic scoping review

Table 1. PubMed final search string

(01st January 1996 - 31st January 2022): (((((((((physical activity[Title/Abstract]) OR (sport*[Title/Abstract])) OR (weight management[Title/Abstract])) OR (diet[Title/Abstract])) OR (nutrition[Title/Abstract])) OR (overweight[Title/Abstract])) OR (obesity[Title/Abstract])) AND (interven*[Title/Abstract])) OR (program*[Title/Abstract])) AND (social return on investment[Title/Abstract])

Table 2: Exclusion criteria

**Exclusion screening criteria (title/abstract)**	
**1**	Not focussed specifically on one of the following key themes: weight management, overweight and/ or obesity, sedentary behaviour, lifestyle, sports/ physical activity, nutrition/ diet
**2**	Not focussed on Social Return on Investment (SROI)
**3**	Not from a high-income country (according to the World Bank[1])
**4**	Not available in the English language
**5**	Only protocol available, no findings/ results of study
**Exclusion eligibility criteria (full text)**	
**6**	No data (description) available of the social (environmental or economic) return/ value created by the intervention identified
**7**	No secondary analysis only primary data sources (no systematic reviews for example)
**8**	Return or savings measured using methods others than SROI
**9**	No physical activity, diet, weight management related outcome
**10**	Not focussed on children, adolescence, or adulthood but infancy stage

Table 3. 12-point quality assessment framework for SROI studies[2]

	**Dimension**	**Criterion**
I	Transparency about why SROI was chosen (“referring to how strong the study relates to the broader impact discourse and how much background information it gives on the method - is fairly well advanced”)	• Linked to context discussion?
II	Documentation of the analysis (published full and summary report available to get an insight of the comprehensive analysis to avoid misinterpretation and establish legitimacy of the method)	• Analysis well documented?
		• Impact map used?
III	Study design (approximation of ‘dead-weight’) (elements of ex-ante and ex-post observations included)	• Control group setup applied?
		• Ex ante- ex post observations performed?
	Precision of the analysis	• Indicators valid and comprehensive?

[2] Krlev, G., Münscher, R., & Mülbert, K. (2013). Social Return on Investment (SROI): state-of-the-art and perspectives-a meta-analysis of practice in Social Return on Investment (SROI) studies published 2002-2012. Social Return on Investment (SROI): state-of-the-art and perspectives - a meta-analysis of practice in Social Return on Investment (SROI) studies published 2002-2012 - heiDOK (uni-heidelberg.de)

IV	(capture of social effects, which is needed to be enhanced in both quantitative and qualitative ways)	• Proxies valid and comprehensive?
		• Social effects captured? (qualitatively)
		• Social effects captured? (quantitatively)
V	Reflection of the results	• Limitations discussed?
		• SROI ratio interpreted?
		• Sensitivity analysis performed?

One point is given to each criterion "present" in the study and 0 if the item was "missing" or "could not be ascertained". According to the framework Krlev et al. describe 70% as a "good score", classifying papers into high quality, if the study scored ≥ 70 % and low quality, if the study scored < 70 %.

Table 7. Outcomes

Physical activity interventions	
**Reference**	**Outcomes**
Oshimi D, Yamaguchi S, Fukuhara T, Tagami Y. (2022). [38]	- Development of children's exercise motivation<br>- Fostering parents' attention to Matsumoto Yamaga Football Club (MYFC)<br>- Improvement of coaching skill<br>- improvement of teaching skills<br>- Reduction of teachers' labour load<br>- promotion of soccer/ physical activity in their region<br>- local governments' labour reduction to provide sports opportunities
Davies LE., Taylor P, Ramchandani G., Christy E. (2021). [39]	General participation<br>- Improved health<br>- Reduced crime<br>- Improved educational performance<br>- Enhanced human capital<br>- Improved subjective wellbeing<br><br>Physical Activity Referral Scheme<br>- Improved physical health<br>- Improved mental health<br>- Confidence<br>- Happiness<br>- Improved relationships with others
Davies, L. E., Taylor, P., Ramchandani, G., Christy E. (2019).[40]	- Improved health (e.g. CHS & stroke, cancer, type 2 diabetes)<br>- Reduced crime (criminal incidences)<br>- Improved educational performance (educational attainment)<br>- Enhanced human capital<br>- Improved subjective well-being (life satisfaction)<br>- Enhanced human resources (non-market benefits)<br><br>**(Eleven social outcomes were identified, which were empirically linked with participation and volunteering in sport. There were six health-related outcomes; two education-related outcomes; and three others related to subjective well-being, crime and the human resource benefits to sports organisations utilising volunteers).*
KPMG Sustainability Consulting Co. (2021). [45]	Lanyu baseball camp and off-site training CTBC Bank<br>- Enhance the image and value of the organization<br>- Improve communication and coordination skills<br>- Increase senses of satisfaction and achievement<br>- Improved interpersonal skills<br><br>Get win Advertising<br>- Increased identification with the organization<br>- Improved communication and coordination skills<br>- Increased sense of satisfaction and achievement

	- Improved interpersonal skills<br><br>High School (coaches)<br>- Increased identification with the organization<br>- Improved communication and coordination skills<br>- Increased sense of satisfaction and achievement<br>- Improved interpersonal skills<br>- Improved skills and abilities to teach basketball<br><br>High School<br>- Increased senses of satisfaction and achievement<br><br>Students<br>- Increased a sense of belonging to the team and the school<br>- Boostered self confidence<br>- Improved discipline and competence<br>- Improved interpersonal skills<br>- Learned baseball knowledge and improve baseball skills<br>- Increased frequency of daily exercise and improve health conditions<br><br>Competing teams<br>- Finance the school's image and popularity<br>- Increased school cohesion<br>- Increased identification with the organization<br>- Increased senses of satisfaction and achievement<br>- Improved interpersonal skills<br>- Improved skills and abilities to teach baseball<br>- Increased a sense of belonging to the team and the school<br>- Boosted self-confidence<br>- Improved discipline and competence<br>- Developed an interest in baseball and enjoy the happiness it brought<br>- Learned baseball knowledge and improve baseball skills<br><br>Fans (family, friends and classmates of players)<br>- Strengthened the relationships with family members and friends<br>- Developed an interest in baseball and enjoy the happiness it brought<br>- Learned baseball knowledge and improve baseball skills
Whitebarn Consulting (2019). [42]	- Improved health<br>- Friendships<br>- Sense of belonging<br>- Relative satisfaction with volunteering for club<br>- Improved skills<br>- Sense of being good parent<br>- Relative satisfaction with having club in local area<br>- Increased profit<br>- Satisfaction with club's contribution<br>- Successful events<br>- Gaelic games and culture are preserved and grown
Baker, C., Courtney, P., Kubinakova, K., Ellis, L., Loughren, E.A. and Crone, D., 2017. [43]	Community connections and resources<br>(Ensuring a stronger future by attracting new members and funding; A greater sense of independence for people of all ages, and better connected people building stronger, safer and more cohesive communities with a clearer sense of community spirit)<br><br>- Improved well-being through development of cultural, recreational and sports facilities<br>- Improved access to community resources<br>- Greater integration of social, sport and special interest groups

	- Improved social capital, community ties and strengthened civic engagement<br><br>Education and skills<br>(More people leading healthier lives and learning new skills; Creating sustainable and innovative ways of doing things in order to achieve greater success)<br>- Increased agency and self-awareness<br>- Reduced social isolation<br>- Improved competence, engagement and purpose<br>- Improved physical, social and life skills and training<br><br>Health and well-being<br>(Fewer barriers and more inclusive opportunities for people to take part in physical activity and sport)<br>- Improved mental health<br>- Safer and more positive environments<br>- Stronger and more connected people and communities<br>- Reduction in chronic disease, LTC and medication<br>- Reduced burden on social care services<br>- Improved physical health and vitality<br>- Improved personal resilience and self-esteem
Charlton C (2014). [44]	- Vitality<br>- Self-esteem<br>- Emotional well-being<br>- Meaning and purpose<br>- Satisfying life<br>- Trust and belonging<br>- Improved physical health (reduced obesity)<br>- Competence<br>- Resilience<br>- Engagement<br>- Autonomy<br>- Supportive relationships
Davies L., Christy E., Ramchandani G., Taylor P. (2021). [46]	The Base Model and the Forecast Model estimate the value of 16 social outcomes:<br>- Physical and mental health (reduced risk of coronary heart disease and stroke; breast cancer; colon cancer; Type 2 diabetes; dementia, clinical depression; improved good health); improved back pain; reduced hip fractures, increased sports injuries);<br>- Mental wellbeing (improved subjective wellbeing/life satisfaction)<br>- Individual development (improved educational attainment and enhanced human capital)<br>- Social and community development (reduced criminal incidences; enhanced social capital and the non-market benefits acquired by organisations utilising sports volunteers
Butler W, Leathem K. (2014). [47]	Young people<br>- Improved wellbeing<br>- Developing higher level sports skills/ gaining sports qualifications<br>- Improved health<br>- Less or more involved in school work<br>- Improved relations with family members<br>- Reduced substance misuse leading too reduced involvement in crime<br>- Improved employability<br>- Gaining employment<br><br>Peers and siblings

	- Reduced involvement in crime<br><br>Volunteers<br>- Increased maturity (better social circle)<br>- More active in locally based positive activities/ keeping out of trouble<br>- Improved skills and employability<br>- Improved sense of personal well-being<br><br>Wider community<br>- Improved and safer living environment for my children<br><br>Partner agencies<br>- Savings as a result of resources not required<br>- Savings as a result of partners working together and not duplicating resources<br>- Other young people services better informed and improving own practice as a result of partnership working<br>- Expertise from ACN - ability to pull in additional funding that would not have happened without the partnership<br><br>State agencies<br>- Police service (cost savings from reduced numbers of young people involved in crimes)<br>- Judicial system (reduced cases being tracked through the court system)<br>- National Health Service (cost savings from the improved health and fitness of young<br>- People Reaching recognised fitness levels through sport)<br>- Department of work and pension (reduced job seekers allowance claims)<br>- Social services (reduced deployment social worker time; family services)
ICF GHK Consulting (2012). [48]	- Increased participation in sport 3 months after project completed (sustained)<br>- Improved fitness and physical health<br>- Increased self-esteem / confidence<br>- Decreased anti-social behaviour<br>- Improved qualifications and opportunities for coaches<br>- Increased use of leisure facilities<br>- Increased sports club membership<br>- Increased capacity for project deliverers to work with commissioning agendas and PBR system
Tilly (2013). [49]	- Improved mental health<br>- Improved physical health<br>- Reduced costs to access provision<br>- Increased employment
New Economics Foundation (2014). [50]	Better health and wellbeing:<br>- Motivation to join the gym<br>- Increased fitness<br>- Better mental wellbeing<br>- Reduced isolation<br><br>Increased employability<br>- Increased skills and knowledge<br>- Increased confidence
Ireland N. (2012). [51]	Service user A (significant improvement in independence and mental health)

	- Improved confidence, self-esteem, sleep patterns, activity levels, reduced stress<br>- Increasing social activity with Gardening in Mind and then elsewhere<br>- Change in physical activity<br>- Eating more healthily<br>- More active<br>- More knowledgeable<br>- Giving something back to the community<br>- Economic activity<br><br>Service user B (managing and improving mental health)<br>- Improvements in confidence and self-esteem<br>- Increasing social activity within Gardening in Mind and elsewhere<br>- Eating more healthily<br>- More active<br><br>Service user C (high risk of substantial relapse/ deterioration without project)<br>- Improvements in confidence and self esteem<br>- Increasing social activity within Gardening in Mind and elsewhere<br>- More active<br><br>Service user D (little sustained impact/ relapse)<br>- Social activity<br><br>Families of service users<br>- Respite for family of service user<br><br>Coventry and Warwickshire Mind<br>- Additional monitoring and care of clients<br><br>NHS community mental health services<br>- Reduction in GP visits<br>- Reduced visits to hospital/ consultant<br>- Reduced medication<br>- Reduction support workers times<br>- Reduced requirement for care packages<br>- Reduction in crisis intervention<br>- Reduction in days respite care to allow families time off<br><br>Benefits provider<br>- Saving in benefits costs as Service User became named carer for mother
Cathay Life Insurance, PricewaterhouseCoopers (2020). [52]	- Better physical heath<br>- Company image improved<br>- Better business performance<br>- Negative impression on Cathay Life generated<br>- Improved interpersonal relationships<br>- Increased stress<br>- Stress relief<br>- Greater convenience in life<br>- Improved job satisfaction<br>- Improved interpersonal relationships<br>- Improved digital application abilities<br>- Risk management awareness enhanced<br>- Greater convenience in life<br>- Vendor income enhanced<br>- Vendor income

E O'Neill (2009). [53]	Health outcomes that might be anticipated from the Greenlink project, based on the available information, are:<br>• Reduced obesity from exercise and healthy eating<br>• Reductions in cancers from reduced obesity and healthy eating<br>• Reduced CVD, stroke and hypertension from reduced obesity and healthy eating-<br><br>Regular volunteers:<br>- Giving something back to the community<br>- Learning new environmental skills<br>- Increased physical health/ stamina<br>- Improved well-being and positive thinking from being outdoors<br>- Making new friends<br>- Meeting new people from the community<br>- Gaining more confidence<br>- Better tolerance of children and young people<br><br>Volunteers on formal training programmes:<br>- Getting a job<br>- More likely to get a job in the future<br><br>Volunteers with learning difficulties<br>- Maintaining skills by having something to do<br><br>Young volunteers<br>- Better tolerance of other generations<br><br>Local residents<br>- Play outside with friends more<br>- Fun events to go to<br>- More cycling and walking<br><br>Local residents – adults:<br>- Nice place to live<br>- More physically active<br>- Friendlier community<br>- Fun events to go to<br><br>CSFT:<br>- Enhanced PR<br>- Enhanced sustainability<br>- Improved learning for the organisation<br>- Higher profile in North Lanarkshire<br><br>North Lanarkshire Council – Regeneration services:<br>- Changed perception in NCL of greenspace as asset of communities<br><br>North Lanarkshire – social services:<br>- Replacement of lost horticultural outdoor activity for clients<br><br>Forestry Commission Scotland:<br>- Opening up woodlands to public access<br>- Safer woodlands<br>- Demonstration of importance of community involvement<br>- Improved biodiversity<br><br>Motherwell police:<br>- Less antisocial behaviour<br><br>The National Health Service:

	- Healthier children<br>- Healthier adults<br>- More activities for people encouraging physical activity
Carrick K. (2013). [54]	NHS Greater Glasgow and Clyde:<br>- Cost savings to the NHS arising as a result of 37 individuals taking part in the programme on a regular basis reducing their blood pressure and requiring less drug therapies<br>- Cost savings to the NHS arising as a result of 60 individuals with clinically diagnosed mental health conditions taking part in the programme on a regular basis and requiring less drug therapies<br><br>Glasgow City Council:<br>- Value in reduced demand for home care as walkers have better mobility, and fewer falls due to increased agility and movement<br><br>Paths for All<br>- Cost savings/As a result of the walks PfA can recruit, train and retain volunteer walk leaders who provide services that would otherwise have to be provided by paid staff.<br>- Volunteers acquire the requisite experience by participating in the walk and this allows PFA to expand the scope of the programme as it is possible to use savings to provide more walking opportunities<br><br>Walk Leaders:<br>- Walk leaders have more social contacts and are now more confident, experience less isolation and take part in new experiences<br>- Walk leaders are much fitter and have improved health as a result of becoming more regularly physically active<br>- Walk leaders have improved self-esteem and a sense of worth as they feel valued by the community<br>- Walk leaders have undertaken and achieved Walk Leader and First Aid certificates. This has increased their levels of confidence and given them practical skills (administration, planning, team working, first aid etc.) to enhance employability or volunteering prospects<br><br>Walkers and Walk Leaders:<br>- Walkers who have experienced mental health problems are able to engage in physical activity and feel happier and positive<br>- Walker with diagnosed physical medical conditions are able to engage in physical activity and as a result feel fitter and become healthier<br><br>Walkers in open walks:<br><br>- Walkers are able to interact with others from different cultural and social backgrounds and to gain a better understanding of ethnicity and disability<br>- Walkers are fitter and have improved physical health as a result of becoming more regularly physically active<br>- Walkers have more social contacts and are now more confident, experience less isolation and take part in new experiences<br>- Walkers are able to be participate in a supported programme that encourages them to progress and achieve a greater sense of personal satisfaction<br>- Walkers form close relationships with friends made on walks after experiencing social isolation after losing partners, retiring or moving into the area

	- Walkers feel safe and comfortable and are able to take part in outdoor physical activity in their local green space by being part of a supported group<br>- Walkers know more about their local area and find new places to visit which increases the sense of satisfaction and enjoyment they derive from the place in which they live<br><br>Walkers in closed Walks:<br><br>- Walkers are fitter and have improved physical health as a result of becoming more regularly physically active<br>- Walkers who are extremely socially isolated and excluded are able to meet people and form new independent friendships<br>- Walkers are able to be participate in a supported programme that encourages them to a progress and achieve a greater sense of personal satisfaction<br>- Walkers are more relaxed and calm and have improved relationships with staff who are able to offer better care and support
Davies L (2018). [55]	Health outcomes<br>- Reduced risk of: CHD and stroke, type 2 diabetes, breast cancer, colon cancer, dementia, clinical depression, good health<br><br>Subjective well-being<br>- Improved subjective well-being for participants and volunteers<br><br>Social capital<br>- Improved social capital for communities<br><br>Education<br>- Improved educational attainment and enhanced human capital<br><br>Crime<br>- Reduced criminal incidences<br><br>Non-market benefits acquired by sports organisations utilising volunteers
Parker KR (2019). [56]	- Improvement in physical health<br>- More energy<br>- Made friends<br>- Have less illness (reduction in medication and use less health services including GP)<br>- Positive effect on mental health
**Nutrition interventions**	
**Reference (ID)**	**Outcomes**
EY Taiwan's Climate Change and Sustainability Services (CCaSS) (2020). [57]	Schoolchildren<br>- Maintenance of health<br>- Enhancement of learning<br>- Self-exploration and development<br>- Improvement of life skills<br><br>Parents<br>- Economizing on breakfast costs<br>- Relieving parental stress<br><br>Teachers<br>- Reducing work-related stress in teachers

	Schools and local communities<br>- Increased income of community economics<br>- Increased connection of community resources
**Cross-cutting interventions**	
**Reference (ID)**	**Outcomes**
Oosterhoff M. et al. (2020) [41]	- Children's Health-related quality of life (HRQOL)<br>- Medical resource use<br>- Families Health-related quality of life (HRQOL)<br>- Absenteeism from school<br>- Opportunities for parents to engage in work/ activities<br>- Parental leave and absenteeism due to sickness of the child
Jones M (2012). [58]	Adult clients<br>- Has lost weight<br>- Is more physically active<br>- Feels more mobile and flexible<br>- Feels overall improved mental wellbeing (improved mood, less anxious, more confident and so forth)<br>- Eats more healthier foods<br>- Makes nee friends/ acquaintances and/ or has more contact with existing social network<br>- Feels personal and social strains associated with lifestyle commitments<br><br>Adult clients (unemployed)<br>- Feels happier, more confident and able to take on care role<br><br>Adult clients (carers of adults)<br>- Child has lost weight<br><br>Children<br>- Feels happier and has improved family life and relationships<br><br>Clients (children)<br>- Child eats more healthy foods<br>- Child feels social and personal strain associated with lifestyle commitments<br>- Better friendships and peer relationships<br>- Reduced specialist consultations (e.g. dieticians)<br><br>Employers of adult clients<br>- Client feels healthier, happier, more confident and able to take up paid work<br><br>NHS Hospital Trust<br>- Reduced hospital interventions<br><br>NHS Primary care/ GP services<br>- Reduced primary care nurse consultations<br>- Client felt overall improved mental wellbeing (improved mood, less anxious, more confident and so forth)<br>- Long term reduced medical costs
